# Lysosomal defects in *Grn* loss of function modulate microglial cell state and immune responses

**DOI:** 10.1002/alz.090872

**Published:** 2025-01-03

**Authors:** Leon Tejwani, Gilbert Di Paolo

**Affiliations:** ^1^ Denali Therapeutics Inc., South San Francisco, CA USA

## Abstract

**Background:**

Macrophages and microglia are myeloid cells that play critical roles in the surveillance of the local environment of the tissues in which they reside. The ability of these phagocytes to perform key functions is contingent on their capacity to sense extracellular cues and mount responses that involve chemotaxis, proliferation, cytokine secretion, and phagocytosis of various cargos for lysosomal clearance. Our overarching hypothesis is that lysosomal degradation of phagocytic cargoes is critical for the resolution of cellular/tissue damage, as well as of inflammation, and that failure to accomplish this step affects myeloid cell states and immune responses.

**Method:**

To test this hypothesis, we examined the consequences of disrupting lysosomal function on myeloid cell state, using GRN deficiency as a model of primary lysosomal dysfunction.

**Result:**

GRN is highly expressed in these cells and encodes progranulin, a frontotemporal dementia‐linked protein critical for the regulation of lysosomal degradative processes. We first performed single‐nucleus RNA‐sequencing of purified microglia from aged wild‐type and Grn knockout (KO) mice, defining the diverse subpopulations that emerge with disease in this context. Next, we characterized primary microglia and macrophages from Grn KO mice cultured in the absence of other cell types, which revealed robust cell autonomous transcriptional and functional alterations resulting from Grn deficiency. To further examine how specific forms of lysosomal dysfunction impact myeloid cell state, we induced pharmacological and genetic perturbations of various lysosomal properties, which uncovered a robust response of myeloid cells to lysosomal deacidification that largely overlaps with that observed in Grn KO myeloid cells. Finally, we identified putative transcription factors that mediate the transcriptional remodeling of myeloid cells in response to lysosomal stress.

**Conclusion:**

Overall, these data reveal a novel link between lysosomal health and myeloid cell transcriptional and functional states.

